# Supplementary material for: Travel surveillance uncovers dengue virus dynamics and introductions in the Caribbean
Source: Nat Commun. 2024 Apr 25;15:3508. doi: 10.1038/s41467-024-47774-8 (PMC11045810; doi:10.1038/s41467-024-47774-8)
Supplement: Supplementary file 1 — Supplementary Information [file 41467_2024_47774_MOESM1_ESM.pdf]

Supplementary Material

Travel surveillance uncovers dengue virus dynamics and introductions in the Caribbean

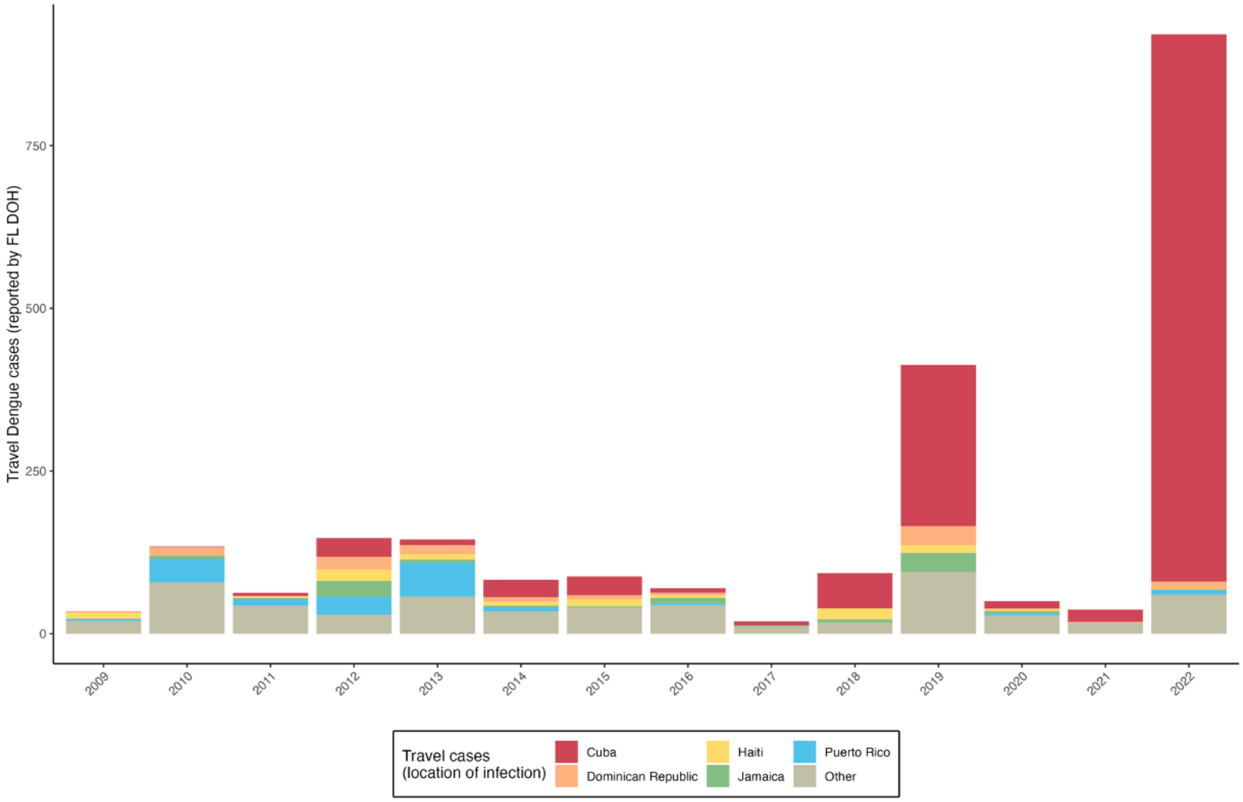

Supplementary Figure 1. Total number of travel-associated dengue cases reported in Florida from 2009 to 2022. Travel-associated dengue cases per year for the top five locations of travel/exposure shown in **Figure 1**. Travel cases from locations outside the top five were combined into the “Other” category. Overall, there were 1283 travel cases from Cuba (57.4%), 106 cases from Dominican Republic (4.7%), 85 cases from Haiti (3.8%), 90 cases from Jamaica (4.0%) and 149 cases from Puerto Rico (6.5%).

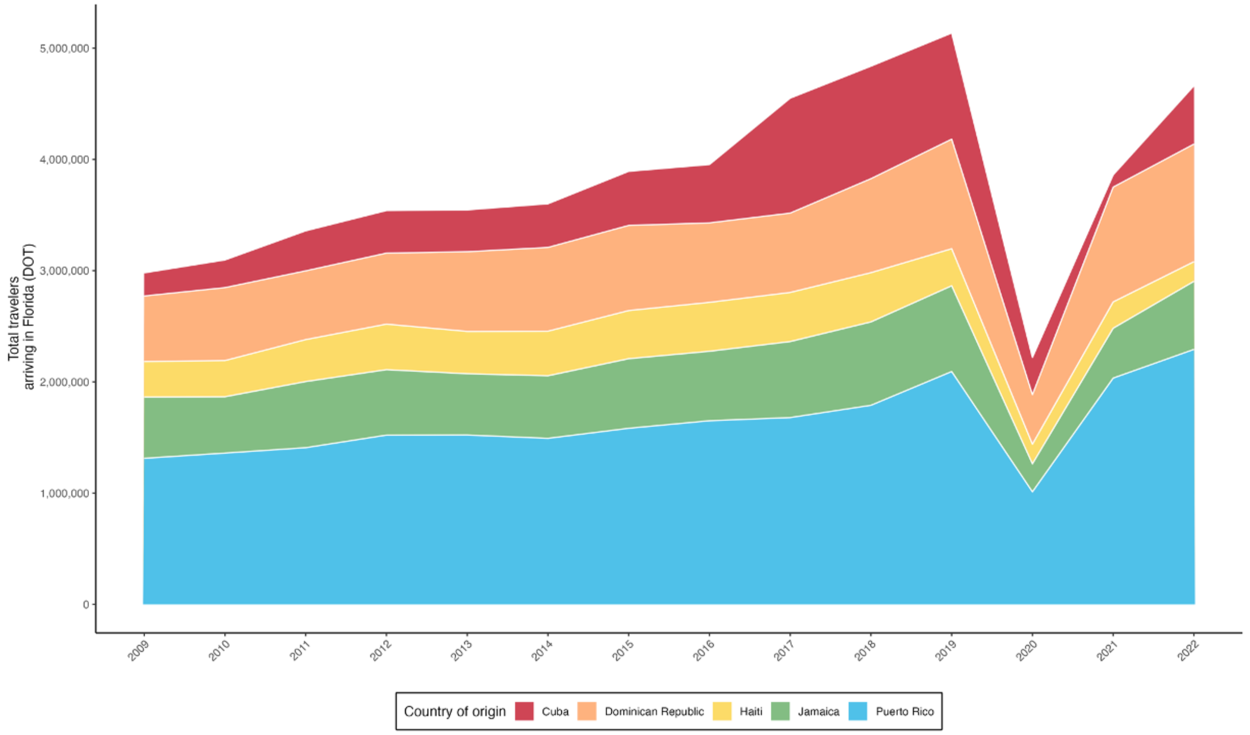

Supplementary Figure 2. Total number of air passenger journeys from Caribbean countries and territories into Florida from 2009 to 2022. Total number of air passenger journeys reported by the United States Department of Transportation (DOT) from the top five locations of travel/exposure shown in **Figure 1**. These data were used to calculate the dengue travel infection rates shown in **Figure 2** and used in the model in **Figure 3**. Air travel was low in 2020 due to COVID-19 pandemic travel restrictions.

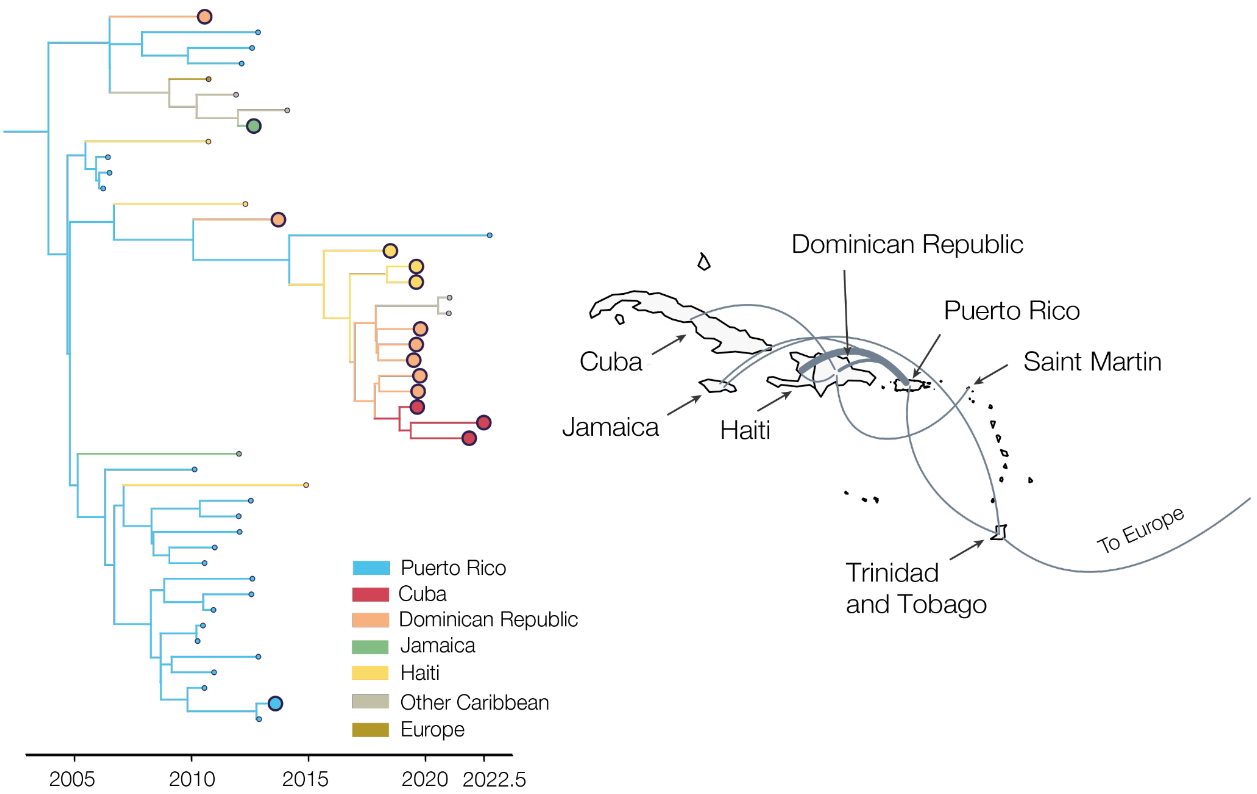

Supplementary Figure 3. Within-Caribbean spread of a DENV-2 clade. Time-scaled subtree of DENV-2 colored by inferred and sampled locations. Shows transitions from Puerto Rico, mostly to the Dominican Republic, and further onto Haiti and Cuba. Map shows the region, with "Other Caribbean" movements going directly to the islands in question.

Supplementary Table 1. Total travel-associated dengue cases reported by the FDOH from 2009-2022, broken down by country or territory, year and serotype. Serotype data was not available for all cases. For Cuba in 2019 and 2022, both the total number of cases and number of cases first identified through syndromic surveillance are included.

County of Likely Exposure	Year	DENV-1	DENV-2	DENV-3	DENV-4	Mixed	Total Cases (syndromic)
Angola	2013	1					1
Antigua and Barbuda	2019			1			1
Antigua and Barbuda	2020			1			1
Aruba	2011						1
Asia	2019	1					1
Bahamas	2020	1					1
Bahamas	2011	8					13
Bangladesh	2013	1	1				3
Bangladesh	2015	1					1
Bangladesh	2010						1
Bangladesh	2011			1			3
Bangladesh	2014						1
Bangladesh	2018						1
Bangladesh	2019			1			1
Bangladesh	2021			1			1
Bangladesh	2022			1			2
Barbados	2013						1
Belize	2019		2				2
Belize/Cuba/Honduras/Mexico	2019		1				1
Bhutan/India	2017			1			1
Bolivia	2020	2					4
Bolivia	2009						2
Bolivia	2013						1
Bolivia	2014				1		1
Brazil	2011	1					4
Brazil	2013	1			1		4
Brazil	2017	1					1
Brazil	2014	2					2
Brazil	2015	2			1		5
Brazil	2016	4					4
Brazil	2022	7					9
Brazil	2009						1
Brazil	2010						1

Brazil	2019		2				3
Brazil	2020		1				1
Brazil	2021						1
Cambodia/Thailand/Vietnam	2019	1					1
Caribbean	2013	1			1		2
Caribbean	2014						1
Caribbean	2022			1			1
Caribbean/Mexico	2021		1				1
Cayman Islands	2010						1
Cayman Islands/Mexico	2019		1				1
Central America	2022			1			1
Central America/Mexico	2019						1
Colombia	2011	1					1
Colombia	2016	1	1		2		4
Colombia	2018	1					1
Colombia	2021	1		1			5
Colombia	2020	2					2
Colombia	2022	2					2
Colombia	2009						2
Colombia	2010						8
Colombia	2012						1
Colombia	2013		1	1			5
Colombia	2014						1
Colombia	2015						1
Colombia	2019			1			2
Colombia/Venezuela	2019						1
Costa Rica	2015	1					3
Costa Rica	2022	1					2
Costa Rica	2016	2	1				9
Costa Rica	2014	3					5
Costa Rica	2010						4
Costa Rica	2011						1
Costa Rica	2013			1			4
Costa Rica	2019		2				2
Cuba	2013	1	2	1	2		9
Cuba	2014	3	5	2	1		27
Cuba	2020	6	1	1			11
Cuba	2021	8	5	1		1	19
Cuba	2019	18	192			3	248 (18)

Cuba	2022	43	91	550	49	7	848 (397)
Cuba	2010						1
Cuba	2011				2		5
Cuba	2012			1	17	1	29
Cuba	2015		5	11		1	29
Cuba	2016			3	2		7
Cuba	2017		6				6
Cuba	2018		41				54
Cuba/Bahamas	2014		1				1
Cuba/Central America	2022	1					3
Cuba/Jamaica	2019			1			1
Cuba/Mexico	2017	1					1
Dominica	2013				1		1
Dominica	2019			2			3
Dominican Republic	2014	1	2		2		7
Dominican Republic	2015	1	2				6
Dominican Republic	2022	1	9				11
Dominican Republic	2010	2	1				13
Dominican Republic	2020	2					2
Dominican Republic	2013	6					14
Dominican Republic	2012	7	2		1		19
Dominican Republic	2019	24	1				29
Dominican Republic	2009						3
Dominican Republic	2011		1				1
Dominican Republic	2016						4
Dominican Republic	2021		1				1
Ecuador	2012	1			1		4
Ecuador	2021	2					2
Ecuador	2010						1
El Salvador	2012	1					2
El Salvador	2022	1			1		2
El Salvador	2010						1
El Salvador	2013						1
El Salvador	2014						2
El Salvador	2015						1
El Salvador	2016						1
El Salvador	2019		2				3
French Polynesia	2020		1				1
Ghana	2012						1

Ghana	2016			1			1
Grenada	2011	1					1
Grenada	2010						4
Guadeloupe	2014						1
Guatemala	2022	2	1				4
Guatemala	2009						2
Guatemala	2010						2
Guatemala	2012						1
Guatemala	2014						1
Guatemala	2015						1
Guatemala	2016						1
Guatemala	2017		1				1
Guatemala	2019		1	2			3
Guatemala	2021				1		1
Guyana	2011						1
Guyana	2012						2
Guyana	2014				1		1
Guyana	2022			1			1
Haiti	2011	1					2
Haiti	2016	1	1		1		4
Haiti	2022	1					1
Haiti	2013	2			2		8
Haiti	2014	2					6
Haiti	2012	6			1		18
Haiti	2019	7	1				12
Haiti	2018	15					17
Haiti	2009						9
Haiti	2010		1				6
Haiti	2015		4		2		11
Haiti	2020		2				2
Hawaii	2015	2					3
Honduras	2019	2	10	1			18
Honduras	2009						2
Honduras	2010		2				6
Honduras	2012		1				1
Honduras	2013		1				5
Honduras	2014		3	1		2	8
Honduras	2015						1
Honduras	2016		1				1

Honduras	2021				1		2
Honduras	2022				1		2
India	2013	1					1
India	2015	1			1		3
India	2016	1		1			4
India	2009						3
India	2011						1
India	2012						1
India	2017		3				3
India	2018		1	1			3
India	2019		3	3	1		10
India	2022		3	1			5
India/Malaysia	2019						1
India/Vietnam	2019						1
Indonesia	2013			1			1
Indonesia	2016		1				1
Indonesia	2017						1
Indonesia	2020						2
Indonesia	2022						1
Indonesia/Malaysia/Singapore	2019		1				1
Jamaica	2011	1					2
Jamaica	2013	3					5
Jamaica	2012	11	1				24
Jamaica	2010		1				5
Jamaica	2014						2
Jamaica	2015						1
Jamaica	2016			6	1		9
Jamaica	2017		1	1			2
Jamaica	2018		1	3			5
Jamaica	2019		2	20		1	29
Jamaica	2020			3			4
Jamaica	2022			1			1
Malaysia	2009						1
Malaysia/Dubai/Bangladesh	2010						1
Malaysia/Thailand	2016						1
Maldives	2022		1				1
Martinique	2010	1					2
Mexico	2013	1					2
Mexico	2016	1					1

Mexico	2021	1					1
Mexico	2014	2					3
Mexico	2019	2	3				6
Mexico	2022	6	3	1			11
Mexico	2009						1
Mexico	2010		1				1
Mexico	2012						1
Mexico	2015				1		3
Mexico	2018						2
Mexico	2020						2
Mexico/Cuba	2020	1					1
Montserrat	2012	1					1
Nicaragua	2010	1		2			15
Nicaragua	2012	1					2
Nicaragua	2013	1					3
Nicaragua	2009						1
Nicaragua	2011						2
Nicaragua	2015				1		3
Nicaragua	2016						3
Nicaragua	2019		13			1	17
Nicaragua	2020		1				1
Nicaragua	2022				4		6
Nigeria	2013	1					2
Nigeria	2017						1
Nigeria	2018			1			1
Pakistan	2016	1		1			2
Pakistan	2022	2		1			3
Pakistan	2010		1				1
Pakistan	2011						1
Pakistan	2017			1			1
Pakistan	2021		1				1
Panama	2011	1	1				2
Panama	2013	1					3
Panama	2022	1					2
Panama	2019	2					1
Panama	2009						3
Panama	2012						1
Paraguay	2020				2		2
Peru	2021						1

Philippines	2012	1			1		3
Philippines	2009						1
Philippines	2010						1
Philippines	2013				1		2
Philippines	2015						4
Philippines	2016						1
Philippines	2017			1			1
Philippines	2018			1			1
Philippines	2019		2				3
Portugal	2012						1
Puerto Rico	2020	1					3
Puerto Rico	2011	5					10
Puerto Rico	2010	6	3		1		36
Puerto Rico	2022	6					7
Puerto Rico	2012	13			2	1	28
Puerto Rico	2013	27			10		52
Puerto Rico	2009						3
Puerto Rico	2014		1		1		7
Puerto Rico	2015		1				1
Puerto Rico	2016						2
Puerto Rico/Mexico	2012	1					1
Puerto Rico/US Virgin Islands	2020	1					1
Singapore	2013	1					1
Sri Lanka	2013				1		1
Sri Lanka	2016						1
Sri Lanka	2012	1					1
Sri Lanka	2011	2					2
St. Barthelemy	2014						1
St. Barthelemy	2022			1			2
St. Barthelemy	2016	1					1
St. Lucia	2020	1	1				3
St. Lucia	2021	1					1
St. Maarten	2011	1					2
St. Martin	2020			1			1
St. Martin	2020	1					1
St. Martin/ St. Barthelemy	2020	1					1
St. Vincent and the Grenadines	2012	1					1
Suriname	2009						1
Thailand	2019	1	2				3

Thailand	2010						1
Thailand	2015						1
Thailand	2016						2
Thailand	2018		1				1
Thailand	2020						1
Trinidad and Tobago	2014	1			1		2
Trinidad and Tobago	2010						1
Trinidad and Tobago	2011				1		5
Trinidad and Tobago	2012						2
Trinidad and Tobago	2013						1
Turks and Caicos	2011						1
Uruguay	2022						1
US Virgin Islands	2012	1					1
US Virgin Islands	2015	1					1
US Virgin Islands	2010						2
US Virgin Islands	2013						3
US Virgin Islands	2018			1			1
US Virgin Islands	2020		1				2
US/British Virgin Islands	2010						1
Venezuela	2010	1		1	1		12
Venezuela	2014	1					3
Venezuela	2013	2		3	1		9
Venezuela	2015	2	1	1			8
Venezuela	2018	4	1				6
Venezuela	2019	5					5
Venezuela	2011						1
Venezuela	2012						1
Venezuela	2016		1	1			6
Vietnam	2011	1					1
Vietnam	2019	2					2
Vietnam	2015						1

Supplementary Table 2 Number of sequences in phylogeographic discrete trait analysis by region and serotype obtained from GenBank and after down-sampling.

Region	DENV-1	DENV-2	DENV-3	DENV-4
Africa	43	78	16	2
Central America	84	109	47	9
Cuba	22	108	165	18
Dominican Republic	7	19	0	3
Eastern Mediterranean	8	30	9	1
Europe	6	2	0	0
Haiti	6	3	0	5
Jamaica	3	3	10	1
North America	1	5	0	0
Other Caribbean	19	12	11	2
Puerto Rico	55	199	108	45
South America	212	217	159	55
South East Asia	220	258	182	117
Western Pacific	409	363	132	87

Supplementary Table 3 Number of sequences from travelers by country or territory and serotype sequenced in this study.

Country	DENV-1	DENV-2	DENV-3	DENV-4
Bolivia	2	0	0	1
Brazil	3	0	0	0
Cambodia	1	0	0	0
Columbia	1	1	1	0
Costa Rica	1	1	0	0
Cuba	21	88	96	14
Dominica	0	0	0	1
Dominican Republic	7	2	0	1
El Salvador	0	0	0	1
Guatemala	0	1	0	0
Haiti	3	1	0	1
Honduras	1	4	0	0
India	0	3	0	0
Jamaica	1	0	10	1
Mexico	2	3	0	0
Nicaragua	0	7	0	2
Paraguay	0	0	0	1
Philippines	0	1	0	0
Puerto Rico	1	2	0	4
Saint Lucia	0	0	1	0
Venezuela	2	2	0	0
