# Supplementary material for: Travel surveillance uncovers dengue virus dynamics and introductions in the Caribbean
Source: Nat Commun. 2024 Apr 25;15:3508. doi: 10.1038/s41467-024-47774-8 (PMC11045810; doi:10.1038/s41467-024-47774-8)
Supplement: Supplementary file 3 — Reporting Summary [file 41467_2024_47774_MOESM3_ESM.pdf]

nature portfolio

Corresponding author(s): Emma Taylor-Salmon, MD & Nathan D Grubaugh, PhD

Last updated by author(s): Apr 4, 2024

Reporting Summary

Nature Portfolio wishes to improve the reproducibility of the work that we publish. This form provides structure for consistency and transparency in reporting. For further information on Nature Portfolio policies, see our Editorial Policies and the Editorial Policy Checklist.

Statistics

For all statistical analyses, confirm that the following items are present in the figure legend, table legend, main text, or Methods section.

n/a	Confirmed	
☐	☒	The exact sample size (n) for each experimental group/condition, given as a discrete number and unit of measurement
☒	☐	A statement on whether measurements were taken from distinct samples or whether the same sample was measured repeatedly
☐	☒	The statistical test(s) used AND whether they are one- or two-sided<br>*Only common tests should be described solely by name; describe more complex techniques in the Methods section.*
☐	☒	A description of all covariates tested
☒	☐	A description of any assumptions or corrections, such as tests of normality and adjustment for multiple comparisons
☐	☒	A full description of the statistical parameters including central tendency (e.g. means) or other basic estimates (e.g. regression coefficient) AND variation (e.g. standard deviation) or associated estimates of uncertainty (e.g. confidence intervals)
☐	☒	For null hypothesis testing, the test statistic (e.g. F, t, r) with confidence intervals, effect sizes, degrees of freedom and P value noted<br>*Give P values as exact values whenever suitable.*
☐	☒	For Bayesian analysis, information on the choice of priors and Markov chain Monte Carlo settings
☒	☐	For hierarchical and complex designs, identification of the appropriate level for tests and full reporting of outcomes
☐	☒	Estimates of effect sizes (e.g. Cohen's d, Pearson's r), indicating how they were calculated

Our web collection on statistics for biologists contains articles on many of the points above.

Software and code

Policy information about availability of computer code

Data collection	No software was used for data collection
Data analysis	We constructed a negative binomial regression model to predict local infection rates from the travel infection rates. All code and model results are available at: https://github.com/grubaughlab/2023_paper_DENV-travelers .

For manuscripts utilizing custom algorithms or software that are central to the research but not yet described in published literature, software must be made available to editors and reviewers. We strongly encourage code deposition in a community repository (e.g. GitHub). See the Nature Portfolio guidelines for submitting code & software for further information.

Data

Policy information about availability of data

All manuscripts must include a data availability statement. This statement should provide the following information, where applicable:

- Accession codes, unique identifiers, or web links for publicly available datasets
- A description of any restrictions on data availability
- For clinical datasets or third party data, please ensure that the statement adheres to our policy

All DENV genomes and sequencing data generated in this study are available on BioProject PRJNA951702. Alignments also include all GenBank accession numbers for any publicly available sequences that were analyzed by not sequenced in this study. Epidemiological data was downloaded from the PAHO website.

The travel surveillance data is available in the Supplementary Information file. The air passenger data used in this study are proprietary and were purchased from OAG Aviation Worldwide Ltd. These data were used under the United States Centers for Disease Control and Prevention license for the current study, and so are not publicly available. The authors are available to share the air passenger data upon reasonable request and with the permission of OAG Aviation Worldwide Ltd.

Research involving human participants, their data, or biological material

Policy information about studies with human participants or human data. See also policy information about sex, gender (identity/presentation), and sexual orientation and race, ethnicity and racism.

Reporting on sex and gender	N/A
Reporting on race, ethnicity, or other socially relevant groupings	N/A
Population characteristics	N/A
Recruitment	N/A
Ethics oversight	The Institutional Review Board (IRB) from the FDOH, Universidad Iberoamerican (UNIBE), Florida Gulf Coast University, and the Yale University Human Research Protection Program detected that pathogen genomic sequencing of de-identified remnant diagnostic samples as conducted in this study is not research involving human subjects (Yale IRB Protocol ID:2000033281; UNIBE-CEI#2021-88).

Note that full information on the approval of the study protocol must also be provided in the manuscript.

Field-specific reporting

Please select the one below that is the best fit for your research. If you are not sure, read the appropriate sections before making your selection.

☐ Life sciences ☐ Behavioural & social sciences ☒ Ecological, evolutionary & environmental sciences

For a reference copy of the document with all sections, see nature.com/documents/nr-reporting-summary-flat.pdf

Ecological, evolutionary & environmental sciences study design

All studies must disclose on these points even when the disclosure is negative.

Study description

In this study, we combined genomic and epidemiological data to investigate outbreak dynamics, dengue virus serotype turnover, and patterns of spread within the Caribbean from 2009-2022. During that time, the Florida Department of Health reported between 19 (in 2017) to 929 (in 2022) travel-associated dengue cases per year, for a total of 2,300 cases, with the majority (1,815 cases, 78.9%) occurring in travelers who recently returned from the Caribbean.

Research sample

Our study used existing datasets. We obtained the number of passengers traveling by air from Cuba, the Dominican Republic, Haiti, Jamaica, and Puerto Rico that disembarked in the US port of entry in Florida from 2009 to 2022 as international and domestic non-stop segment traffic reports (T100) from the Office of Data, Analytics, and Technology (DAT), Division of Global Migration Health (DGMH). The flight travel volume data were provided by OAG Aviation Worldwide Ltd. OAG DOT Analyser, T100 International and Domestic Segment Traffic Reports, Version 2.6.1 2023 (https://analytics.oag.com/analyser-client/home; accessed May 16, 2023). United States Department of Transportation (DOT) data includes all commercial chartered and military flights arriving in airports in the United States and includes origin and destination.

Weekly cumulative reports on travel-associated dengue case numbers were collected from 2009 to 2022 and are publicly available from the FDOH. These cases reported on the FDOH database include those that were confirmed by both PCR and serologic assays. Serotype data for travel-associated dengue cases was determined by the FDOH using the CDC DENV-1–4 RT-PCR Assay. Travel-associated cases occurred in individuals who had traveled to a dengue-endemic country or territory in the two weeks prior to symptom onset.

Patient serum samples were selected for study inclusion according to Ct values from prior FDOH Trioplex and serotype-specific PCR diagnostics and/or volume availability. Sample extracts were kept frozen on dry ice during transport and shipped to collaborators for subsequent sequencing. All samples were de-identified to maintain patient privacy and blind the study. The objective of this study was to analyze viral genomic data, so no human data was used.

Infectious disease surveillance of travelers has been shown to supplement local surveillance in low-resource areas. Infected travelers can also be sentinels for pathogen transmission in locations where outbreaks have not yet been reported. Utilizing traveler data could be particularly useful for supplementing surveillance in the Caribbean because of the popularity of tourism in the region. In the US, dengue is the leading cause of febrile illness among travelers returning from the Caribbean. In Florida, the number of travel-associated dengue cases has increased dramatically in recent years, making Florida a good sentinel for the Caribbean, due to its geographic location and high volume of travel back and forth between Florida and the islands.

Sampling strategy

No sample calculation was performed. Sample sizes were determined by the number of travel-associated dengue case numbers reported by the Florida Department of Health (FDOH) in a given year (from 2009-2022). Dengue is a reportable disease in the United

	States. In addition to health care provider and laboratory reporting, the FDOH uses syndromic surveillance to aid in case identification. FDOH detected syndromic surveillance cases by searching de-identified chief complaints, admission and discharge diagnoses, travel-related fields, etc., obtained from participating emergency rooms and urgent care centers in Florida. Information from suspected dengue cases are forwarded to the county health departments, who in turn reach out to the local hospitals and ascertain whether dengue testing was ordered.<br><br>For phylogeographic analyses, the larger and more geographically representative a dataset is, the better. Dengue samples are scarce, and we sampled as many samples as possible.
Data collection	None of the authors were present during sample collection. Samples were collected by healthcare workers in hospitals and sent to Public Health labs. Concerning genomic data, they were generated by MIB, CC, AS, VH, and CBFV, using Illumina platforms.
Timing and spatial scale	Data was collected from January 2009 through December 2022, and samples was collected on a monthly and yearly basis. Data was collected in Florida from infected patients who had traveled to a dengue-endemic area within 2 weeks of symptom onset.
Data exclusions	For this study, we only included patients who traveled to one endemic location within the 2 weeks prior to symptoms onset so we could more accurately sort the temporal and spatial distribution of travel-associated cases. This led to the exclusion of 28 patients who traveled to multiple countries during our study period, constituting 1.2% of the 2,300 total cases. Within the Caribbean, the focus of this paper, there were 18 patients that were excluded due to traveling to multiple countries, constituting 1% of the 1,815 total cases. Therefore, due to the small number of these cases, we did not feel that excluding them would affect our analysis. We aggregated the data by year and by location of likely exposure (i.e., travel origin).<br><br>One limitation of DOT data is that it does not include connecting flight information. However, as this would lead to a potential over-estimation of total travelers and, therefore, potentially an under-estimation of travel infection rates, we felt this was reasonable. Cruise and boat travel data were not included. There were very few infections linked with boat travel in our dataset, even between Cuba and Florida. There were also very few infections linked to cruise travel. It may be that these cases are more likely to be tourists who are subsequently diagnosed elsewhere (and just visiting Florida for the cruise departure) or that they may have different risk behaviors compared to air travelers.<br><br>Enhanced surveillance was increased during 2019 and 2022 due to the large number of travel-associated cases being detected. These efforts involved adding recent travel to Cuba as a criterion, in addition to dengue-like symptoms. Eighteen of the 413 travel-associated cases in 2019 and 397 of the 929 cases in 2022 were first identified via syndromic surveillance and used in our analysis. Of note, 116 cases identified via enhanced surveillance in 2022 would not have met our case definition without this additional criterion. Therefore, in order to counter these differences in case ascertainment, we only included cases captured via traditional reporting, not enhanced detection of cases among travelers returning from Cuba, for our calculations.
Reproducibility	The only experiment performed in this study was genomic sequencing. We followed standard protocols (described in detail in the Methods section), and each genome was sequenced only once, since the average depth of sequencing was large enough to provide good genome coverage (>70% coverage at 10X or higher).
Randomization	No randomization was used as samples were already assigned identifiers based on de-identified ID, host country of remnant sample, or country of travel departure
Blinding	No blinding was performed as sample names were labeled based on de-identified ID, host country of remnant sample, or country of travel departure. No blinding was done to avoid sample cross-contamination.

Did the study involve field work? ☐ Yes ☒ No

Reporting for specific materials, systems and methods

We require information from authors about some types of materials, experimental systems and methods used in many studies. Here, indicate whether each material, system or method listed is relevant to your study. If you are not sure if a list item applies to your research, read the appropriate section before selecting a response.

Materials & experimental systems

n/a	Involved in the study
☒	☐ Antibodies
☒	☐ Eukaryotic cell lines
☒	☐ Palaeontology and archaeology
☒	☐ Animals and other organisms
☒	☐ Clinical data
☒	☐ Dual use research of concern
☒	☐ Plants

Methods

n/a	Involved in the study
☒	☐ ChIP-seq
☒	☐ Flow cytometry
☒	☐ MRI-based neuroimaging

Plants

Seed stocks	*Report on the source of all seed stocks or other plant material used. If applicable, state the seed stock centre and catalogue number. If plant specimens were collected from the field, describe the collection location, date and sampling procedures.*
Novel plant genotypes	*Describe the methods by which all novel plant genotypes were produced. This includes those generated by transgenic approaches, gene editing, chemical/radiation-based mutagenesis and hybridization. For transgenic lines, describe the transformation method, the number of independent lines analyzed and the generation upon which experiments were performed. For gene-edited lines, describe the editor used, the endogenous sequence targeted for editing, the targeting guide RNA sequence (if applicable) and how the editor was applied.*
Authentication	*Describe any authentication procedures for each seed stock used or novel genotype generated. Describe any experiments used to assess the effect of a mutation and, where applicable, how potential secondary effects (e.g. second site T-DNA insertions, mosiacism, off-target gene editing) were examined.*
